# A Five Immune-Related lncRNA Signature as a Prognostic Target for Glioblastoma

**DOI:** 10.3389/fmolb.2021.632837

**Published:** 2021-02-16

**Authors:** Xiaomeng Li, Li Sun, Xue Wang, Nan Wang, Kanghong Xu, Xinquan Jiang, Shuo Xu

**Affiliations:** ^1^ School of Public Health, Shandong First Medical University & Shandong Academy of Medical Sciences, Taian, China; ^2^ Department of Neurosurgery, Qilu Hospital of Shandong University, Jinan, China; ^3^ Brain Science Research Institute, Shandong University, Jinan, China

**Keywords:** glioblastoma, long non-coding RNA, immune, signature, prognostic

## Abstract

**Background:** A variety of regulatory approaches including immune modulation have been explored as approaches to either eradicate antitumor response or induce suppressive mechanism in the glioblastoma microenvironment. Thus, the study of immune-related long noncoding RNA (lncRNA) signature is of great value in the diagnosis, treatment, and prognosis of glioblastoma.

**Methods:** Glioblastoma samples with lncRNA sequencing and corresponding clinical data were acquired from the Cancer Genome Atlas (TCGA) database. Immune-lncRNAs co-expression networks were built to identify immune-related lncRNAs via Pearson correlation. Based on the median risk score acquired in the training set, we divided the samples into high- and low-risk groups and demonstrate the survival prediction ability of the immune-related lncRNA signature. Both principal component analysis (PCA) and gene set enrichment analysis (GSEA) were used for immune state analysis.

**Results:** A cohort of 151 glioblastoma samples and 730 immune-related genes were acquired in this study. A five immune-related lncRNA signature (*AC046143.1, AC021054.1, AC080112.1, MIR222HG*, and *PRKCQ-AS1*) was identified. Compared with patients in the high-risk group, patients in the low-risk group showed a longer overall survival (OS) in the training, validation, and entire TCGA set (*p* = 1.931e-05, *p* = 1.706e-02, and *p* = 3.397e-06, respectively). Additionally, the survival prediction ability of this lncRNA signature was independent of known clinical factors and molecular features. The area under the ROC curve (AUC) and stratified analyses were further performed to verify its optimal survival predictive potency. Of note, the high-and low-risk groups exhibited significantly distinct immune state according to the PCA and GSEA analyses.

**Conclusions:** Our study proposes that a five immune-related lncRNA signature can be utilized as a latent indicator of prognosis and potential therapeutic approach for glioblastoma.

## Introduction

Glioblastoma is the most prevalent and fatal primary brain tumor around the world (McGuire, 2016). Even given the optimal therapeutic approaches combined with surgical resection, targeted radiotherapy, high-dose chemotherapy as well as novel electric field treatment, the median overall survival (OS) is still less than 21 months (Tan et al., 2020; Delgado-Martín and Medina, 2020; Stupp et al., 2017). In the past decades, seminal discoveries have clarified the mechanism of immune response within glioblastoma, and emerging immune therapeutic strategies have exhibited great potential by initiating and amplifying host anti-tumor immunity (Turkowski et al., 2018; Silver et al., 2016; Sampson et al., 2017). However, glioblastoma can hardly be eradicated due to profound tumor-mediated immunosuppression (Jackson et al., 2019; McGranahan et al., 2019). Therefore, immune-related biomarkers of this malignancy do not facilitate the diagnosis and prognosis evaluation but rather offer an extraordinary glimpse of the tumor pathophysiology.

Long noncoding RNA (lncRNA), of which length ≥ 200 bp, exhibited a wide range of regulatory activities without protein-coding capacity (Kopp and Mendell, 2018). Abundant evidence has demonstrated that lncRNAs were extensively expressed in various tumors and involved in tumorigenesis, tumor progression, infiltration, and metastasis (Bhan et al., 2017; Li et al., 2018b; Jiang et al., 2019). In glioblastoma, lncRNA *MALAT1* contributes to tumor proliferation and progression by *MALAT1/miR-199a/ZHX1* axis (Liao et al., 2019). *HOTAIRM1* promotes glioblastoma growth and invasion by up-regulating *HOXA1* Gene (Li et al., 2018a). *ADAMTS9-AS2* triggers temozolomide resistance via upregulating the *FUS/MDM2* axis in glioblastoma cells (Yan et al., 2019). Meanwhile, lncRNAs also play a vital role in immune regulation (Chen et al., 2017; Wang et al., 2019b). For instance, *Lnc-EGFR* generates immunosuppressive status in patients with hepatocellular carcinoma by facilitating regulatory T cell differentiation (Jiang et al., 2017). *Lnc-BHLHE40-AS1* promotes the development of early breast cancer by regulating *STAT3* signaling and builds an immune-permissive microenvironment (DeVaux et al., 2020). Until recently, it was reported that certain immune-related lncRNA signature has predictive potential for the survival of glioma patients ( Xia et al., 2020; Tian et al., 2020; Wang et al., 2020 ), indicating that lncRNAs also participate in the glioma-mediated immune dysregulation within the blood-brain barrier. Nevertheless, the connection between immune-related lncRNAs and prognosis prediction of glioblastoma is worth further exploration.

In the present study, we took advantage of the Cancer Genome Atlas (TCGA) database to establish an immune-related lncRNA signature for glioblastoma patients, which might act as a key prognosis predictor and promising immunotherapeutic targets for this malignancy.

## Materials and Methods

### Data Source

Normalized RNA-seq data with the estimation of Fragments Per Kilobase of exon model per Million mapped fragments (FPKM) of glioblastoma patients and the corresponding clinical information were acquired from TCGA database (https://portal.gdc.cancer.gov/, RRID: SCR_003193). The exclusion criteria were as follows: 1) samples with unknown survival information; 2) samples with OS less than 30 days, who died because of nonneoplastic factors, such as myocardial infarction, hemorrhage, and severe infection (Cheng et al., 2015; Li and Guo, 2020). A total of 151 TCGA glioblastoma samples were included for the subsequent analysis, that were randomly split up into the training set and the validation set. The training set with 76 samples were used to construct the prognosis model. The validation set with 75 samples and the entire TCGA set were then applied to check the results of the training set. The IRB approval and the documentation of informed consent are waived, because all the data acquired from the TCGA database are open to the public.

### Immune-Related lncRNAs

Immune-associated genes associated with glioblastoma were downloaded from the Molecular Signatures Database v7.0 (Immune system process M13664, Immune response M19817, https://www.gsea-msigdb.org/gsea/msigdb/index.jsp, RRID: SCR_016863) (Cheng et al., 2016). In total, 322 immune-related genes were extracted after integration (Subramanian et al., 2005; Cheng et al., 2016). 730 immune-related lncRNAs were then determined based on immune-lncRNAs co-expression network and Pearson correlation analysis (|R| > 0.5, FDR < 0.001).

### Signature Construction

Univariate cox regression analysis was used to identify lncRNAs that were significantly associated with prognosis (*p* < 0.01). Stepwise multivariate Cox regression analysis was employed to develop a risk score. The purpose of the risk score is to determine a uniform signature for subsequent prognostic prediction. The risk score formula was as follows (Cheng et al., 2015; Yang et al., 2019):

$$\mathrm{Risk score}=\beta_{\mathrm{gnen1}}\times \exp r_{\mathrm{gene1}}+\beta_{\mathrm{gnen2}}\times \exp r_{\mathrm{gene2}}+\cdots +\beta_{\mathrm{gnenn}}\times \exp r_{\mathrm{genen}}.$$

Expr_gene_ is prognostic-related lncRNAs expression level, while β refers to multivariate Cox regression model regression coefficient which was computed by log-transformed hazard ratio (HR) in the training set (Lossos et al., 2004). Patients were then divided into high- and low-risk group with median risk score as cut-off value. The signature was verified in the validation set and the entire TCGA set.

### Data Processing

We used the Cytoscape software (version 3.7.2, Cytoscape consortium, RRID: SCR_003032) to build immune-lncRNAs co-expression networks (Shannon et al., 2003). Principal component analysis (PCA) was used to evaluate the expression profile between different groups. We then employed the gene set enrichment analysis (GSEA; https://www.gsea-msigdb.org/gsea/index.jsp, RRID: SCR_016863) to discover statistical differences of grouped samples. Statistical significance was expressed by normalized enrichment score (NES) and false discovery rate (FDR) (Subramanian et al., 2005; Cheng et al., 2016).

### Statistical Analysis

OS comparisons were carried out by Kaplan-Meier analysis and the log-rank test. Independent prognostic factors for glioblastoma were calculated via univariate and multivariate Cox proportional hazards analysis. The HR and 95% confidence intervals (CI) were obtained at the same time. The area under the ROC curve (AUC) and stratification analysis were performed to compare the survival predictive power of the signature. We carried out all the analyses with R program 3.6.2 (http://www.r-project.org, R Project for Statistical Computing, RRID: SCR_001905,). A 2-side *p* value < 0.05 was regarded as statistically significant.

## Results

### Immune-Related lncRNAs in Glioblastoma

322 immune-related genes associated with glioblastoma were downloaded from the Molecular Signatures Database. 730 immune-related lncRNAs were then acquired by immune-lncRNAs co-expression networks and Pearson correlation analysis (|R| > 0.5, FDR < 0.001; Supplementary Figure S1A). 151 TCGA glioblastoma patients were divided into the training set (n = 76) and the validation set (n = 75) randomly and equally. Demographic and clinical features were briefly shown in Supplementary Table S1. Univariate Cox proportional hazards regression analysis was implemented in the training set to screen out the lncRNAs with significant prognostic values. A total of 15 immune-related lncRNAs were identified to correlate to the glioblastoma patients’ survival (*p* < 0.01) and then enrolled into the candidate pool for further analysis. Finally, 5 out of 15 candidate lncRNAs were selected through multivariate Cox proportional hazards regression as independent prognostic factors (Table 1). Among these immune-related lncRNAs, 4 lncRNAs (*AC046143.1*, *AC021054.1*, *AC080112.1*, *MIR222HG*) were categized as deleterious factors on the basis of their β value, whereas *PRKCQ-AS1* tended to be protective and its high expression was associated with longer survival. Immune-lncRNAs co-expression networks were further assessed as shown in Supplementary Figure S1B.

**TABLE 1:** Five lncRNAs selected as prognosis-associated factors.

Ensembl database ID	Gene symbol	HR	95% CI	*p* value	β value
ENSG00000229334.1	AC046143.1	2.183	1.329‒3.587	2.04E-03	0.781
ENSG00000177406.4	AC021054.1	2.242	1.457‒3.449	2.39E-04	0.807
ENSG00000266208.1	AC080112.1	1.672	1.224‒2.284	1.25E-03	0.514
ENSG00000270069.1	MIR222HG	2.060	1.381‒3.073	4.00E-04	0.723
ENSG00000237943.5	PRKCQ-AS1	0.537	0.341‒0.845	7.20E-03	−0.621

### Construction and Verification of Prognostic Risk Model

**FIGURE 1:**
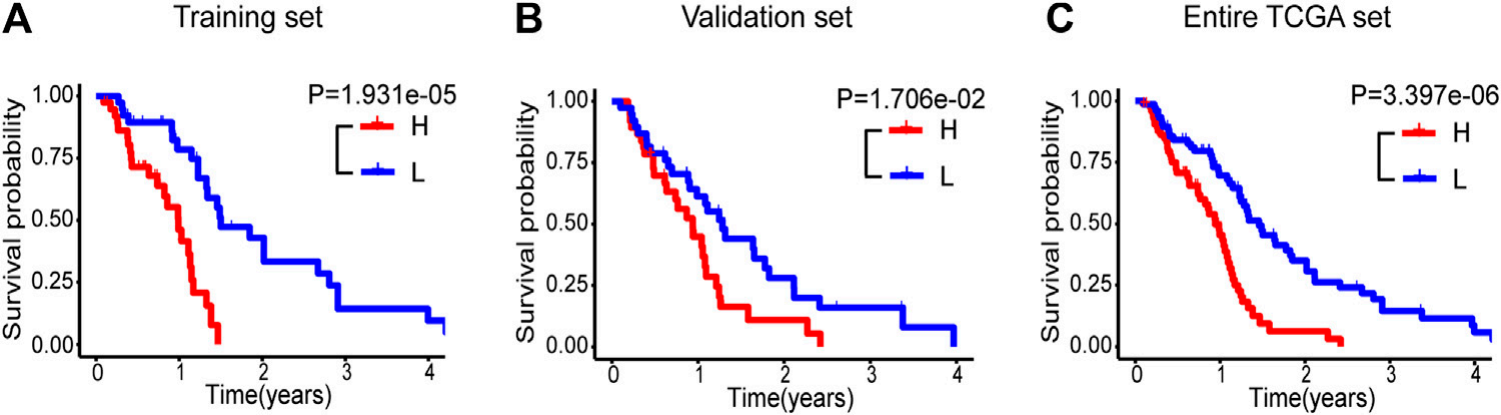
Kaplan-Meier survival curve analyses of OS in high-(red curve) and low-risk (blue curve) group for the training set **(A)**, the validation set **(B)** and the entire TCGA set **(C)**. OS, overall survival.

We hereby employed a risk score approach to construct a five immune-related lncRNA signature (Cheng et al., 2015; Kiran et al., 2019; Gao et al., 2018). With the median risk score as a cut-off value, samples in the training set of glioblastoma patients were divided into high-(n = 38) and low-risk (n = 38) groups. There was a significant difference in OS between the two groups. Patients with high-risk displayed reduced OS in contrast to those with low-risk (median OS: 0.986 vs 1.488 years, *p* = 1.931e‐05, Figure 1A). To confirm the predictive performance, the identical algorithm and coefficient were then employed in the validation and entire TCGA set, and similar results were obtained from both sets (median OS: 0.903 vs 1.244 years, P=0.017; median OS: 0.939 vs 1.457 years, *p* = 3.397e-06; Figures 1B,C).

**FIGURE 2:**
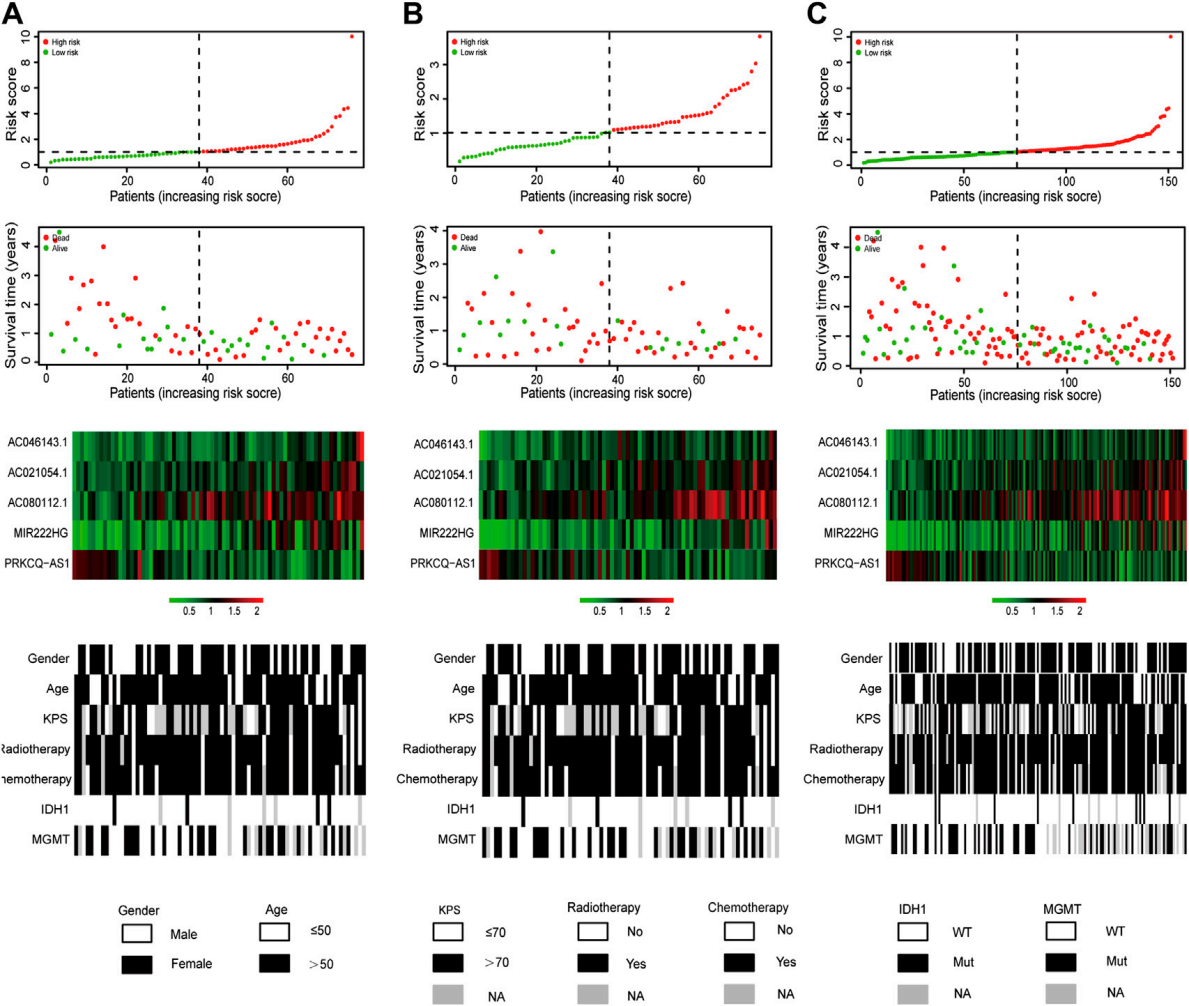
Risk score distribution, survival status, expression of lncRNA, as well as distributed patterns of clinical and molecular characteristics for glioblastoma patients in the training set **(A)**, the validation set **(B)** and the entire TCGA set **(C)**.

As shown in Figure 2A, the risk scores of glioblastoma patients in the training set were sorted, and their survival status were plotted in dot graph. The heat map generated based on the RNAseq data exhibited the distributed expression modes of lncRNA among the two groups. For samples with low risk, the deleterious lncRNAs had relatively low expression except for the protective *PRKCQ-AS1*, which were opposite to the high-risk samples. Distribution tendency among age, treatment options, and molecular features was also conducted. Similar findings were confirmed in the validation set and entire TCGA set (Figures 2B,C). The detailed immune-related lncRNA expression was shown in Supplementary Figure S2.

### The Prognostic Independence of the Five Immune-Related lncRNA Signature

Univariate and multivariate Cox regression analyses were performed to validate whether this five-lncRNA signature along with the other clinicopathological factors were independent prognostic indicators of the glioblastoma patients. As a result, the prognostic independence of the lncRNA signature was proven for OS in the entire TCGA set (HR = 1.324, *p* = 1.26e-05, Table 2). Analogously, age (HR = 2.267, *p* = 0.001), radiotherapy (HR = 0.332, *p* = 5.42e-05) and chemotherapy (HR = 0.419, *p* = 6.64e-04) were also demonstrated to be independent prognostic factors.

**TABLE 2:** Univariate and multivariate Cox regression analysis of overall survival of GBM patients.

Variables	Univariate analysis			Multivariate analysis		
	HR	95% CI of HR	*p* value	HR	95% CI of HR	*p* value
Training set (n = 76)						
Five-lncRNA signature (high/low)	1.506	1.278‒1.776	1.08e-06	1.385	1.159‒1.654	3.38e-04
Age (>50/≤50)	2.739	1.190‒6.304	0.018	2.549	1.103‒5.891	0.029
Gender (male/female)	0.678	0.377‒1.220	0.195	0.721	0.395‒1.317	0.288
KPS (>70/≤70)	0.404	0.175‒0.936	0.035	0.396	0.164‒0.957	0.04
Radiotherapy (yes/no)	0.288	0.132‒0.631	0.002	0.274	0.124‒0.605	0.001
Chemotherapy (yes/no)	0.258	0.120‒0.556	5.41e-04	0.269	0.124‒0.581	8.37e-04
Validation set (n = 75)						
Five-lncRNA signature (high/low)	1.695	1.194‒2.409	0.003	1.579	1.092‒2.282	0.015
Age (>50/≤50)	1.034	1.009‒1.059	0.008	1.028	1.003‒1.054	0.027
Gender (male/female)	0.972	0.561‒1.686	0.92	1.106	0.623‒1.962	0.731
KPS (>70/≤70)	0.995	0.982‒1.008	0.443	0.992	0.979‒1.006	0.263
Radiotherapy (yes/no)	0.288	0.132‒0.631	0.002	0.274	0.124‒0.605	0.001
Chemotherapy (yes/no)	0.553	0.280‒1.094	0.089	0.590	0.294‒1.182	0.137
Entire TCGA set (n = 151)						
Five-lncRNA signature (high/low)	1.425	1.269‒1.601	2.12e-09	1.324	1.167‒1.501	1.26e-05
Age (>50/≤50)	2.388	1.447‒3.940	6.60e-04	2.267	1.370‒3.750	0.001
Gender (male/female)	0.828	0.558‒1.230	0.35	0.961	0.640‒1.445	0.85
KPS (>70/≤70)	0.992	0.982‒1.002	0.116	0.991	0.981‒1.002	0.095
Radiotherapy (yes/no)	0.289	0.169‒0.492	5.17e-06	0.332	0.194‒0.567	5.42e-05
Chemotherapy (yes/no)	0.406	0.246‒0.668	3.97e-04	0.419	0.254‒0.692	6.64e-04

In addition, the prognostic independence of this five-lncRNA signature was evaluated to certain well-known molecular features of glioblastoma. Unsurprisingly, multivariate Cox regression analysis validated its statistical robustness considering the *IDH1* mutation and *MGMT* promoter methylation status (HR = 1.386, *p* = 3.14e-06, Supplementary Table S2). Moreover, *IDH1* mutation status was also obviously related to OS (HR = 0.336, *p*=0.039).

### Identification of Predicting Performance of the Five Immune-Related lncRNA Signature

**FIGURE 3:**
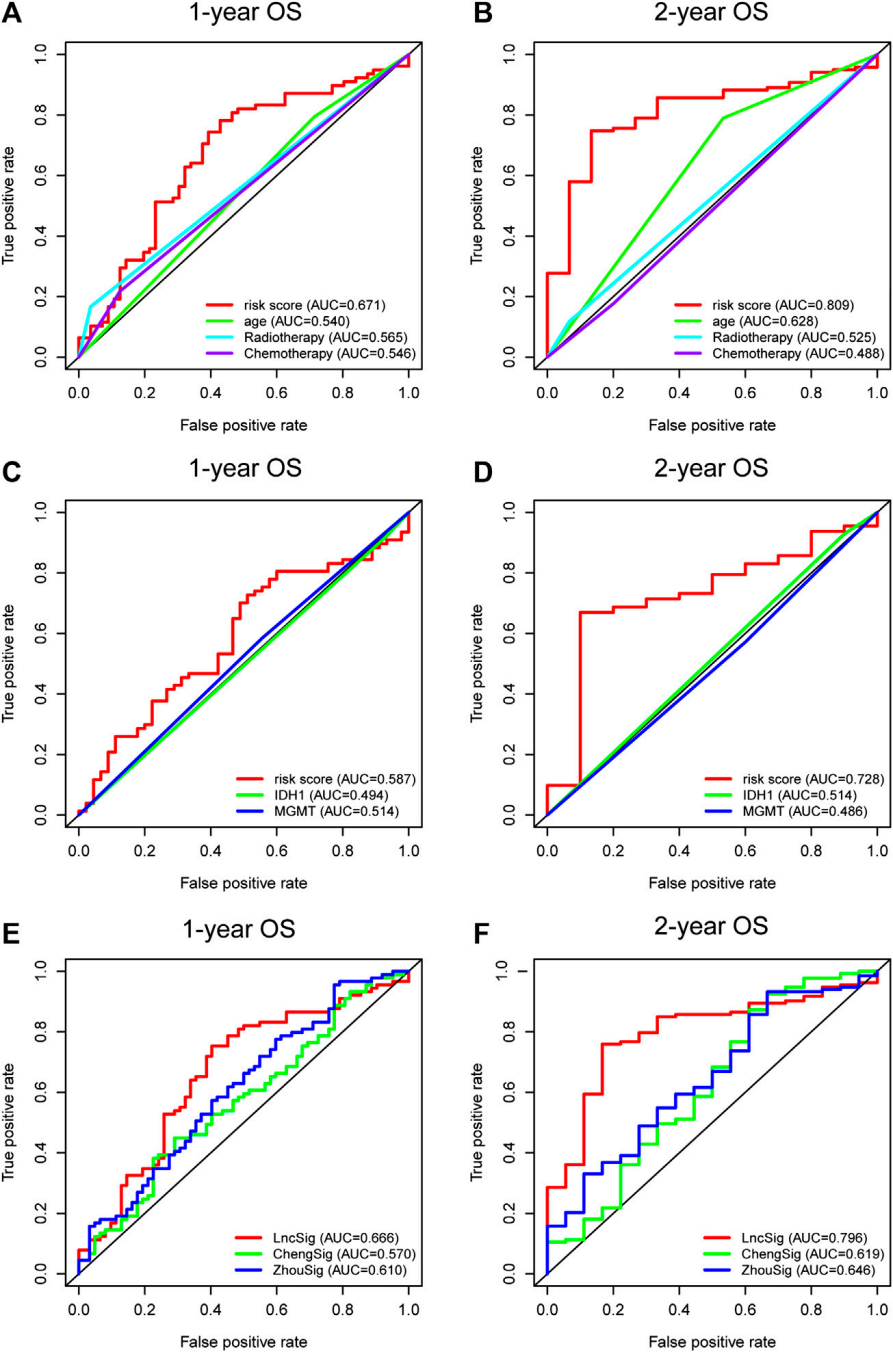
Time-dependent ROC curves of the entire TCGA set indicating the prognostic value of the lncRNA signature and the other clinical independent prognostic factors of 1-year OS **(A)** and 2-year OS **(B)**. The prognostic power of the lncRNA signature and the molecular features of 1-year OS **(C)** and 2-year OS **(D)**. The predictive performance of the lncRNA signature (LncSig) and immune gene signature (ChengSig) and other lncRNA signature (ZhouSig) of 1-year OS (**E**) and 2-year OS (**F**). ROC, receiver operating characteristic; OS, overall survival.

The AUCs were employed to evaluate the predictive accuracy of the prognostic indicator demonstrated by multivariate regression analysis, including age, radiotherapy, chemotherapy, and the immune-related lncRNA signature. The lncRNA signature curve (AUC = 0.671) was higher than that of age (AUC = 0.540), radiotherapy (AUC = 0.565), and chemotherapy (AUC = 0.546) at 1-year OS (Figure 3A). Similar results were demonstrated at 2-year OS (lncRNA signature AUC = 0.809; age AUC = 0.628; radiotherapy AUC = 0.525; chemotherapy AUC = 0.488; Figure 3B). Moreover, we identified its predictive power contrasted to *IDH1* and *MGMT* promotor status. Again, the immune-related lncRNA signature curve showed the greatest AUC value over *IDH1* mutation curve and *MGMT* promoter methylation curve at 1-year OS (lncRNA signature AUC = 0.587; *IDH1* AUC = 0.494; *MGMT* AUC = 0.514; Figure 3C) and 2-year OS (lncRNA signature AUC = 0.728; *IDH1* AUC = 0.514; *MGMT* AUC = 0.486; Figure 3D).

The signature we present, to the best of our knowledge, is the first immune-related lncRNA signature for glioblastoma based on the construction of immune-lncRNAs co-expression networks. However, several other immune-associated signatures have been reported recently to predict the prognosis of glioblastoma patients, including a gene signature reported by Cheng et al. (hereinafter referred to as ChengSig) (Cheng et al., 2016) and a lncRNA signature proposed by Zhou et al. using survival analysis and Cox regression model (hereinafter referred to as ZhouSig) (Zhou et al., 2018). Therefore, we compared the prognostic abilities of our lncRNA signature (hereinafter referred to as LncSig) and the immune-related signature mentioned above. Utilizing the same TCGA patient cohort, LncSig had a better predictive performance at 1-year OS (LncSig AUC = 0.666; ChengSig AUC = 0.570; ZhouSig AUC = 0.610; Figure 3E) and 2-year OS (LncSig AUC = 0.796; ChengSig AUC = 0.619; ZhouSig AUC = 0.646; Figure 3F). In addition, there are several similar immunologically-irrelevant, with which we have also compared, including Zhang (hereinafter referred to as ZhangSig) ( Zhang et al., 2013) and Li’s lncRNA prognostic signature (hereinafter referred to as LiSig) (Li et al., 2019) for glioblastoma and Pan's signature (hereinafter referred to as PanSig) ( Pan et al., 2020) for glioma. Within the same patient cohort, our signature had a better predictive performance at 1-year OS (LncSig AUC = 0.666; ZhangSig AUC = 0.521; LiSig AUC = 0.563; PanSig AUC = 0.491; Supplementary Figure S3A) and 2-year OS (LncSig AUC = 0.796; ZhangSig AUC = 0.548; LiSig AUC = 0.590; PanSig AUC = 0.573; Supplementary Figure S3A).

These data indicated the predictive power of the five immune-related lncRNA signature for the prognosis assessment of glioblastoma.

### Application of the lncRNA Signature in Stratified Groups

**FIGURE 4:**
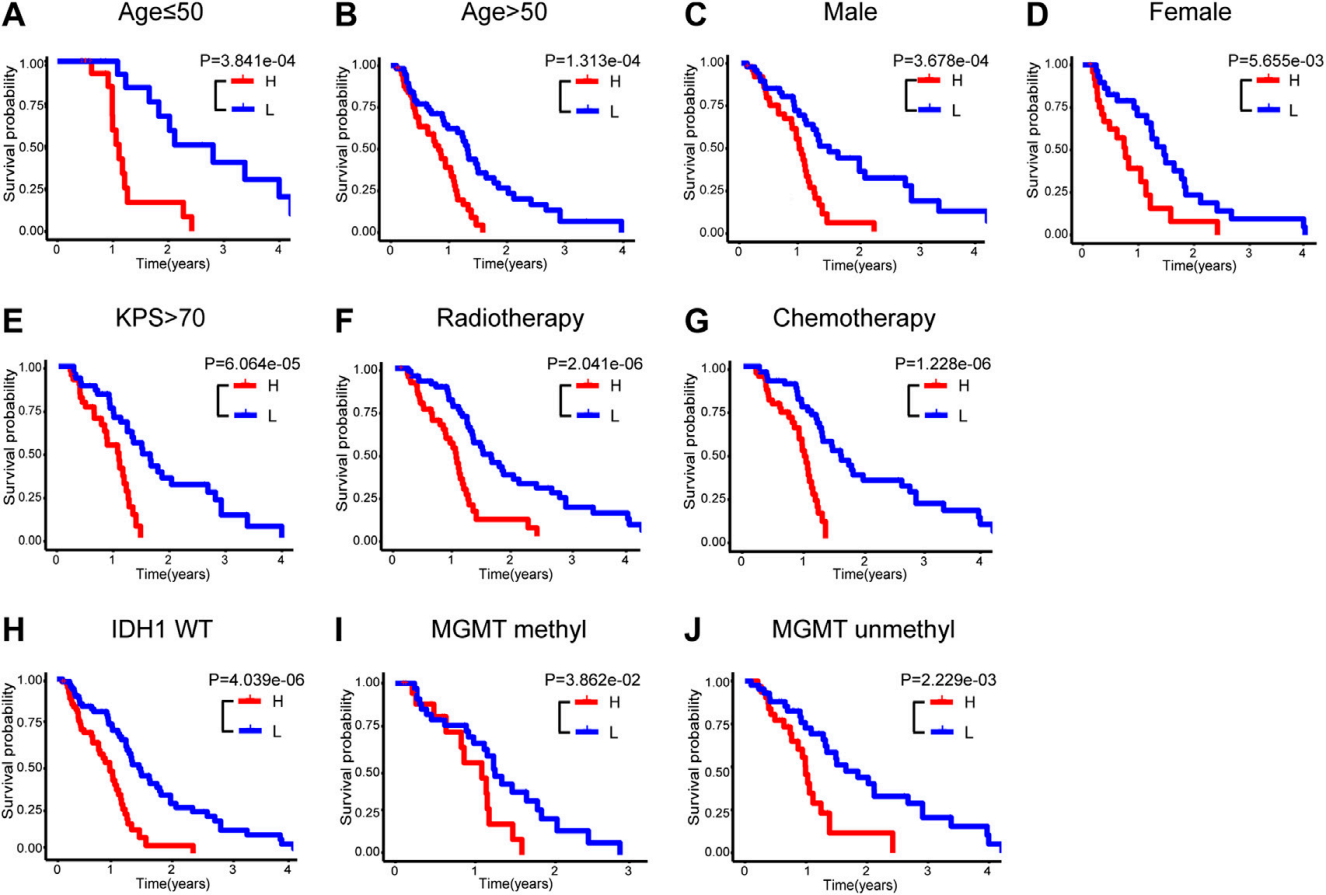
Stratification analyses in the entire TCGA set. Kaplan-Meier survival curve of OS in high- and low-risk groups for the cohorts of age ≤ 50 **(A)**, age > 50 **(B)**, male **(C)**, female **(D)**, high KPS **(E)**, radiotherapy **(F)**, chemotherapy **(G)**, wild-type IDH1 **(H)**, methylated MGMT promoter **(I)** and unmethylated MGMT promoter **(J)**. OS, overall survival; KPS, Karnofsky performance score; IDH, isocitrate dehydrogenase; MGMT, O6-methylguanine-DNA methyltransferase.

To assess the feasibility of this immune-related signature, the glioblastoma patients were further categorized into different stratified groups based on their age, sex, KPS score, radiotherapy and chemotherapy status. Within each group, samples were divided into high- and low-risk groups utilizing the five immune-related lncRNA signature. As shown in Figures 4A–G, patients with lower risk exhibited survival preference in most stratified cohorts consistently, including age ≤ 50 (n = 36, *p* = 3.841e-04), age > 50 (n = 115, *p* = 1.313e-04), male (n = 97, *p* = 3.678e-04), female (n = 54, *p* = 5.655e-03), high KPS (KPS score > 70, n = 83, *p* = 6.064e-05), radiotherapy (n = 126, *p* = 2.041e-06) and chemotherapy (n = 112, *p* = 1.228e-06). Survival analysis also indicated that the patients in the high-risk group had shorter survival in the stratified cohorts of wild-type *IDH1* (n = 136, *p* = 4.039e-06), methylated (n = 52, *p* = 3.862e-02), and unmethylated *MGMT* promoter (n = 73, *p* = 2.229e-03) in Figures 4H–J. These results suggested that this signature acted as a consistently reliable indicator in the assessment of clinical outcomes of glioblastoma patients.

### Immune Status Associated with the lncRNA Signature

**FIGURE 5:**
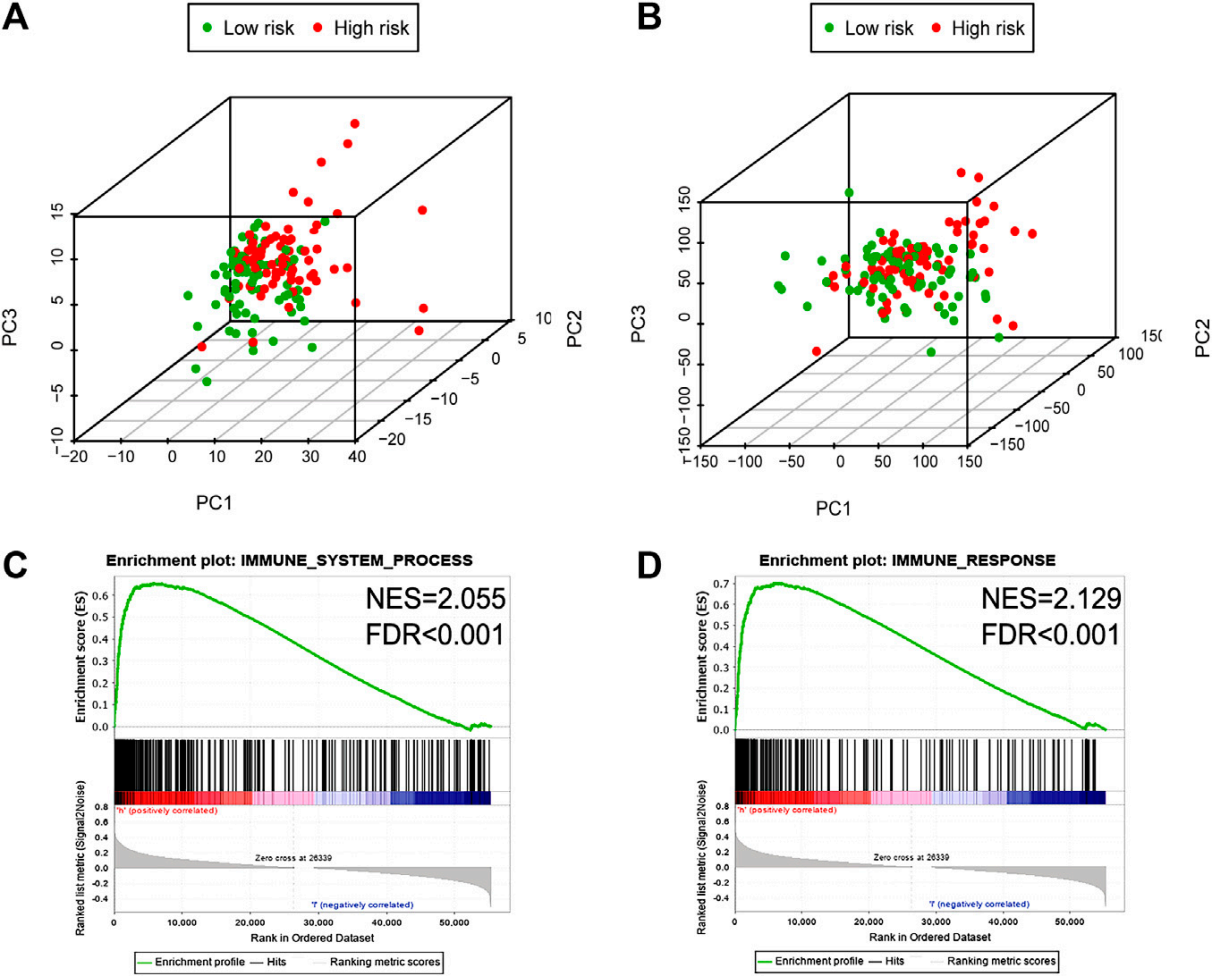
The high-and low-risk group showed different immune state. PCA between high- and low-risk groups on the basis of immune genes **(A)** and whole gene expression profiles **(B)**. GSEA suggested that immune-related phenotypes were highly enriched in the high-risk group **(C, D)**. PCA, Principal components analysis; GSEA, Gene set enrichment analysis.

PCA was conducted to investigate the different distributions based on immune genes and whole-gene expression patterns (Figures 5A,B). The results displayed that the high- and low-risk group tended to be distributed in different directions, indicating that the high-risk group had significant differences in the immune status from the low-risk group. Functional enrichment analysis was then conducted via GSEA (Subramanian et al., 2005; Cheng et al., 2016; Wang et al., 2018). As showed in Figures 5C,D, immune system process and immune response pathways were annotated in the high-risk group in comparison with in the low-risk group. Consequently, there is a close correlation between the signature and the immune status within the glioblastoma environment.

## Discussion

The outcomes for patients with glioblastoma are miserable nowadays, which arouse great efforts to investigate the pathophysiological mysteries of this malignancy. In the past decades, convergent evidence has highlighted that the central nervous system is immunologically distinct rather than privileged (Ransohoff and Engelhardt, 2012; Kipnis, 2016; Engelhardt et al., 2017). However, redundant immunosuppressive mechanisms within the glioblastoma inhibit the anti-cancer immune responses and harness them with tumor-promoting characteristics (Lamano et al., 2019; Tomaszewski et al., 2019). The lncRNA dysregulation has been widely reported in various tumors including glioblastoma (Wang et al., 2019a; Zhuang et al., 2019; Ji et al., 2019). For instance, lncRNA *MIR155HG* was observed that not only associates with poorer OS in glioblastoma but also significantly correlates with infiltrating levels of immune cells and immune molecules (Peng et al., 2019). Consequently, it is necessary for us to study the diagnostic and prognostic values of immune-related lncRNAs for glioblastoma.

In the present study, 151 glioblastoma samples from the TCGA database were enrolled. Immune-lncRNAs co-expression networks and Pearson correlation analysis were conducted to acquire 730 immune-related lncRNAs. A five immune-related lncRNA signature (*AC046143.1*, *AC021054.1*, *AC080112.1*, *MIR222HG*, and *PRKCQ-AS1*) was then developed to classify glioblastoma patients into the high- and low-risk group. Glioblastoma patients in the low risky group showed longer survival time than those in high risky group (*p* = 1.931e-05, *p* = 1.706e-02, and *p* = 3.397e-06, Figure 1). Univariate and multivariate Cox regression analyses further validated the prognostic effect of this five immune-related lncRNA signature, even considering key interference factors such as age, gender, KPS score, radiotherapy, chemotherapy, and molecular features (Table 2; Figure 2). Of note, the AUC of the lncRNA ROC curve was greater than those of any other significant prognostic indicator (Figure 3). Furthermore, this immune-related lncRNA signature consistently discriminated the low risky patients with statistical survival preferences in the most stratified cohorts (Figure 4). Finally, we discovered differences in the immune status associated with the lncRNA signature. Specifically, the samples with high risk tended to display more concentrated immune properties (Figure 5). These findings indicate that this immune-related signature exhibits a vital prognostic effect for glioblastoma.

The mechanisms underlying lncRNA regulation of immune response remain unknown. Among these five enlisted lncRNAs, *PRKCQ-AS1* acts as a protective player, and its high expression is relevant to longer survival. Interestingly, it has been reported that *PRKCQ-AS1* was upregulated in colorectal cancer and associated with reduced survival in colorectal cancer (Shademan et al., 2019), suggesting the complexity and heterogeneity of tumor biology. In contrast, *AC046143.1*, *AC021054.1*, *AC080112.1*, and *MIR222HG* were associated with poor prognosis. There have been quite few studies focusing on these lncRNAs, except that *MIR222HG* expression facilitated the development of castration-resistant prostate cancer (Sun et al., 2018). There were no reports that these identified lncRNAs harbor any immune-related yet; however, immune-lncRNAs co-expression networks in this study indicate that they may regulate immune function either directly or indirectly. For instance, AC021054.1 was shown to be correlated to dozens of downstream genes, which involve the regulations of chemotaxis (CXCR2, CCR5, CCR1, CXCL12) (Gouwy et al., 2014), immune cell infiltration (ITGB2) (Xiu et al., 2020) and phenotype polarization (IL10, IL27RA) (Cox et al., 2011). In addition, we investigated the correlation between these lncRNAs and immune costimulatory molecules with available TCGA data, including CTLA4, PDL1, TIM-3, and B7-H3. Interestingly, AC046143.1 was positively correlated with B7-H3, while the protective factor PRKCQ-AS1 was negatively correlated with B7H3. AC021054.1 was positively correlated with CTLA4 and TIM-3, and MIR222HG was also positively correlated with B7-H3 and PDL1 (Supplementary Figure S4). Moreover, although these lncRNAs are dominantly expressed by glioblastoma, exosomal lncRNA has become elucidated as a key mechanism to abort anti-tumor immunity and induce immunosuppression (Tielking et al., 2019). Thus, our findings could propose several potential lncRNA targets for glioblastoma immunotherapy.

Wang et al. reported a nine immune-related lncRNA signature for patients with anaplastic gliomas, a less malignant form of brain tumor (Wang et al., 2018). Li and Meng also developed an eight immune-related lncRNA signature associated with low-grade glioma prognosis (Li and Meng, 2019). However, these identified lncRNAs are not coincidental with our signature. There could be several reasons. First, their data source and bioinformatic process are quite different. For example, Wang et al. obtained the data from the Chinese Glioma Genome Atlas (CGGA) microarray data, and then validated their findings in two additional datasets (GSE16011, REMBRANDT) but not TCGA as we conducted. Second but more important, all these works, including ours, confirm the complexity of glioma immunity, considering the inconsistent tumorigenesis and regulation characteristics between different histological classifications of gliomas. Meanwhile, Zhou et al. demonstrated that a six-lncRNA signature which improved prognosis prediction was immune-related based on the TCGA data, whose methodology was quite opposite to ours (Zhou et al., 2018). More specifically, they identified their lncRNA signature via Cox regression, and then suggested their signature might be relevant to the immune processes and pathways with in silico functional analysis. In contrast, we screened out the immune-associated lncRNAs by co-expression networks at the beginning, and then identified five lncRNAs with significant prognostic power using a risk score method. Despite the different methodologies, manipulation of glioblastoma immune by various lncRNAs can be demonstrated consistently (Boussiotis and Charest, 2018; Vinay et al., 2015).

Our study has some limitations. First, our lncRNA signature was based on glioblastoma samples acquired from the TCGA data portal. Validation using non-TCGA data, such as CGGA and REMBRANDT, could bring more conviction theoretically; however, it was with considerable difficulty currently because the lncRNA-seq profiling and clinical data varied among different datasets. For instance, the expression profiling of *AC046143.1* was not available in CGGA and REMBRANDT. Second, it’s well-documented that multiple infiltrating immune cells as well as intrinsic tumor cells induce local immune suppression synergistically (Farhood et al., 2019; Heymann and Tacke, 2016). Therefore, it’s urgent to understand how these lncRNAs which are dominantly expressed by glioblastoma modify the phenotype and function of immune cells in the tumor microenvironment. Third, the regulatory mechanisms of these lncRNAs remain largely unknown nowadays. The immune-lncRNAs co-expression network sheds some light on understanding the interactions with some key immune elements; however, the interpretation of this finding should be cautious and further experimental investigation and verification would be necessary. Therefore, we need to conduct further experiments to study the regulatory mechanism of the five lncRNAs in modulating the immune microenvironment within glioblastoma. Despite the limitations mentioned above, our study proposes the very first immune-associated lncRNA signature as a latent prognostic indicator and potential therapeutic approach for glioblastoma.

In conclusion, we identified and verified a five immune-related lncRNA signature (*AC046143.1*, *AC021054.1*, *AC080112.1*, *MIR222HG*, and *PRKCQ-AS1*) which had independent prognostic value for patients with glioblastoma. Therefore, we hope it might be utilized as a potential prognostic indicator and inspire the new immunotherapeutic approach.

## Data Availability

The datasets presented in this study can be found in online repositories. The names of the repository/repositories and accession number(s) can be found in the article/Supplementary Material.

## Abbreviations

AUC, area under curve; CI, confidence intervals; FDR, false discovery rate; GSEA, gene set enrichment analysis; HR, hazard ratio; IDH, isocitrate dehydrogenase; KPKM, fragments per kilobase of exon model per million mapped; KPS, Karnofsky performance score; lncRNA, long noncoding RNA; MGMT, O6-methylguanine-DNA methyltransferase; NES, normalized enrichment score; OS, overall survival; PCA, principal component analysis; ROC, receiver operating characteristic; TCGA, the Cancer Genome Atlas.
