# Supplementary figures and images for: A Five Immune-Related lncRNA Signature as a Prognostic Target for Glioblastoma
Source: Front Mol Biosci. 2021 Feb 16;8:632837. doi: 10.3389/fmolb.2021.632837 (PMC7921698; doi:10.3389/fmolb.2021.632837)

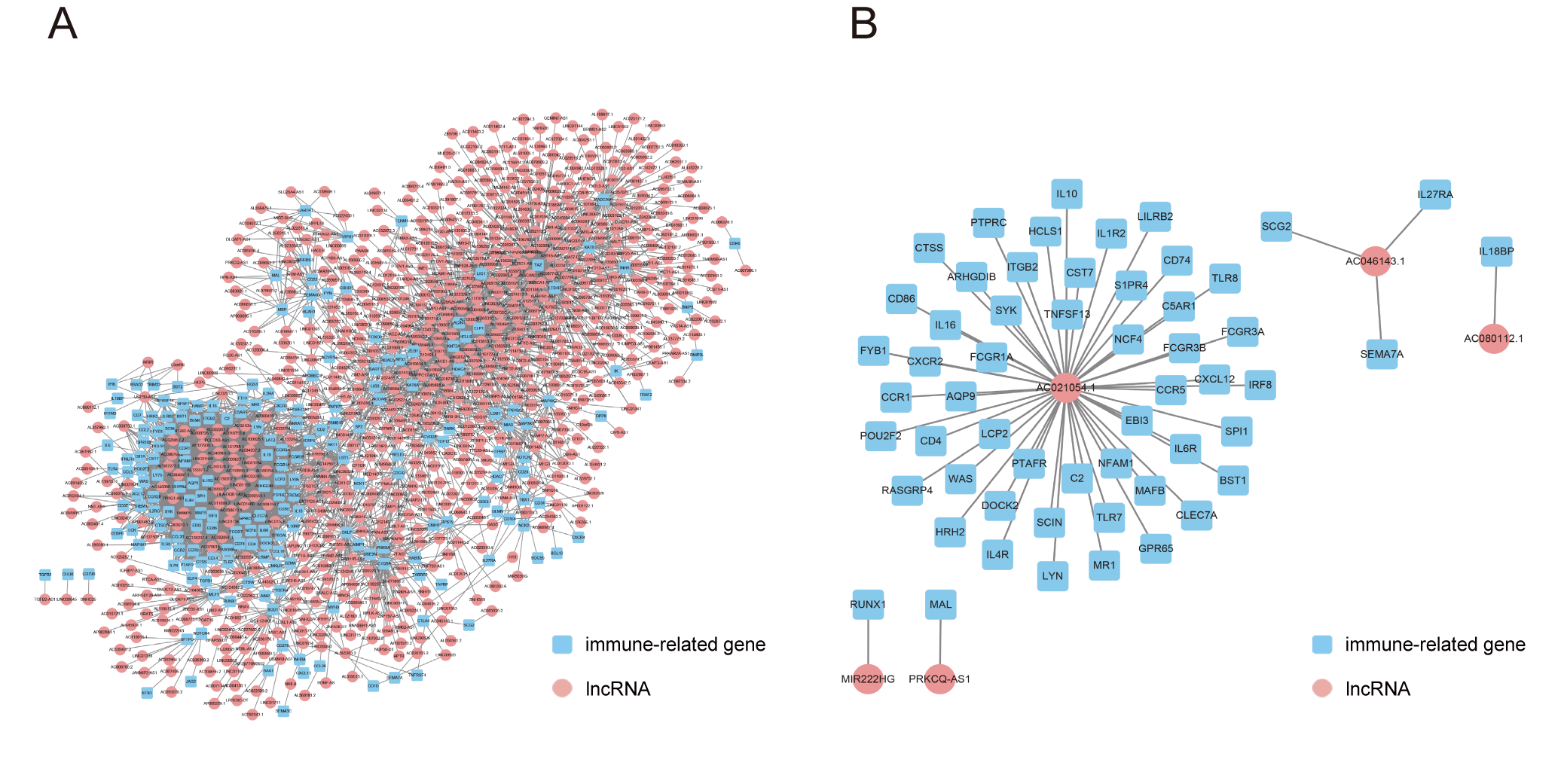

Supplement: Supplementary file 1 [file image1.tif]

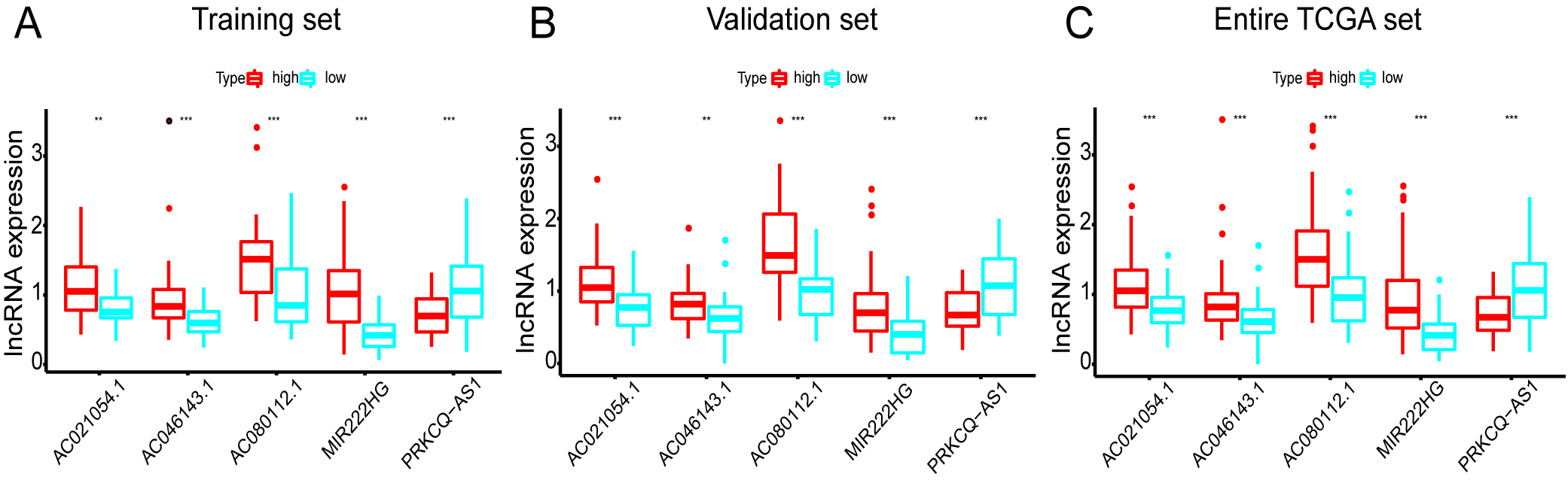

Supplement: Supplementary file 2 [file image2.tif]

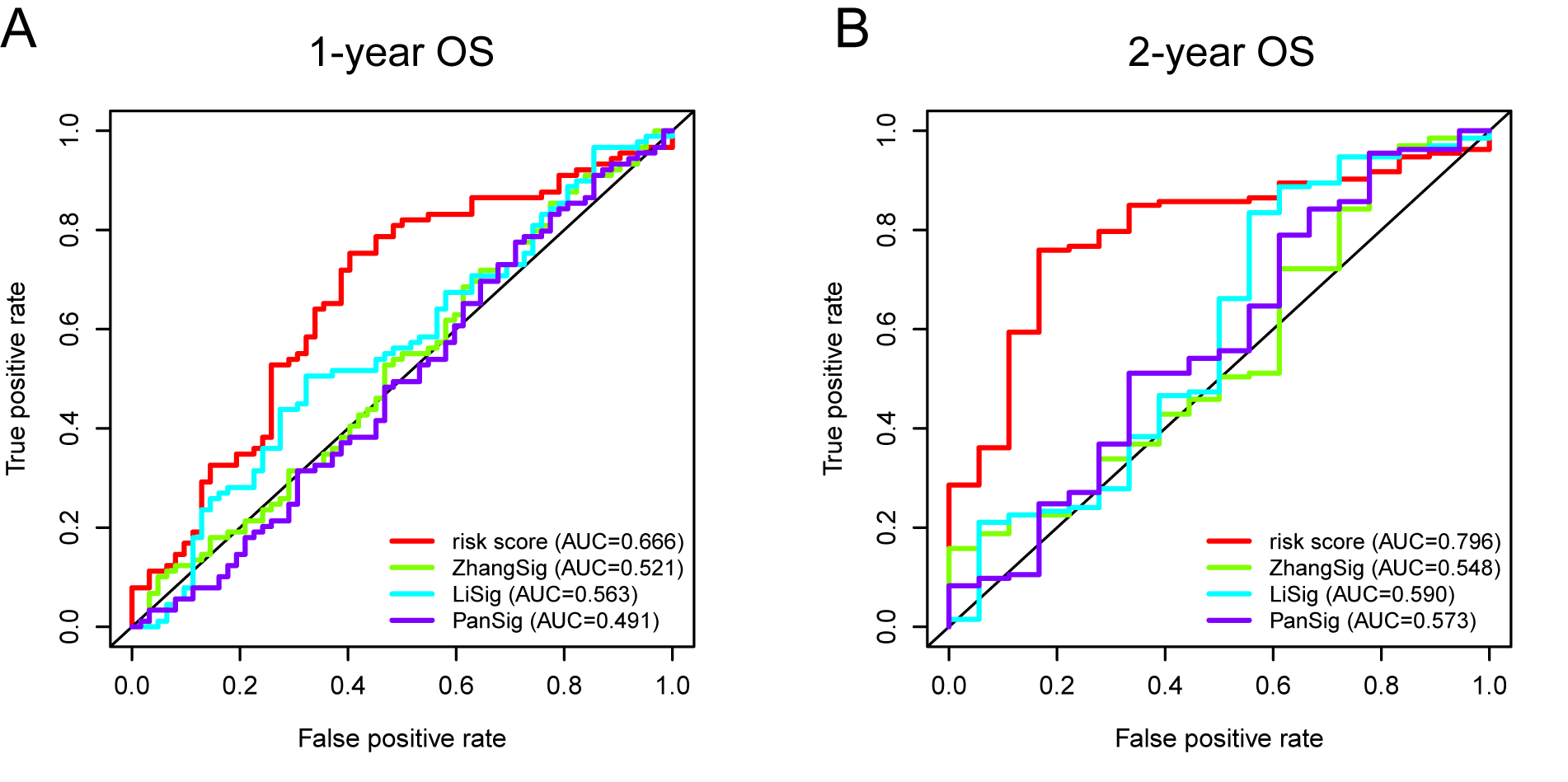

Supplement: Supplementary file 3 [file image3.tif]

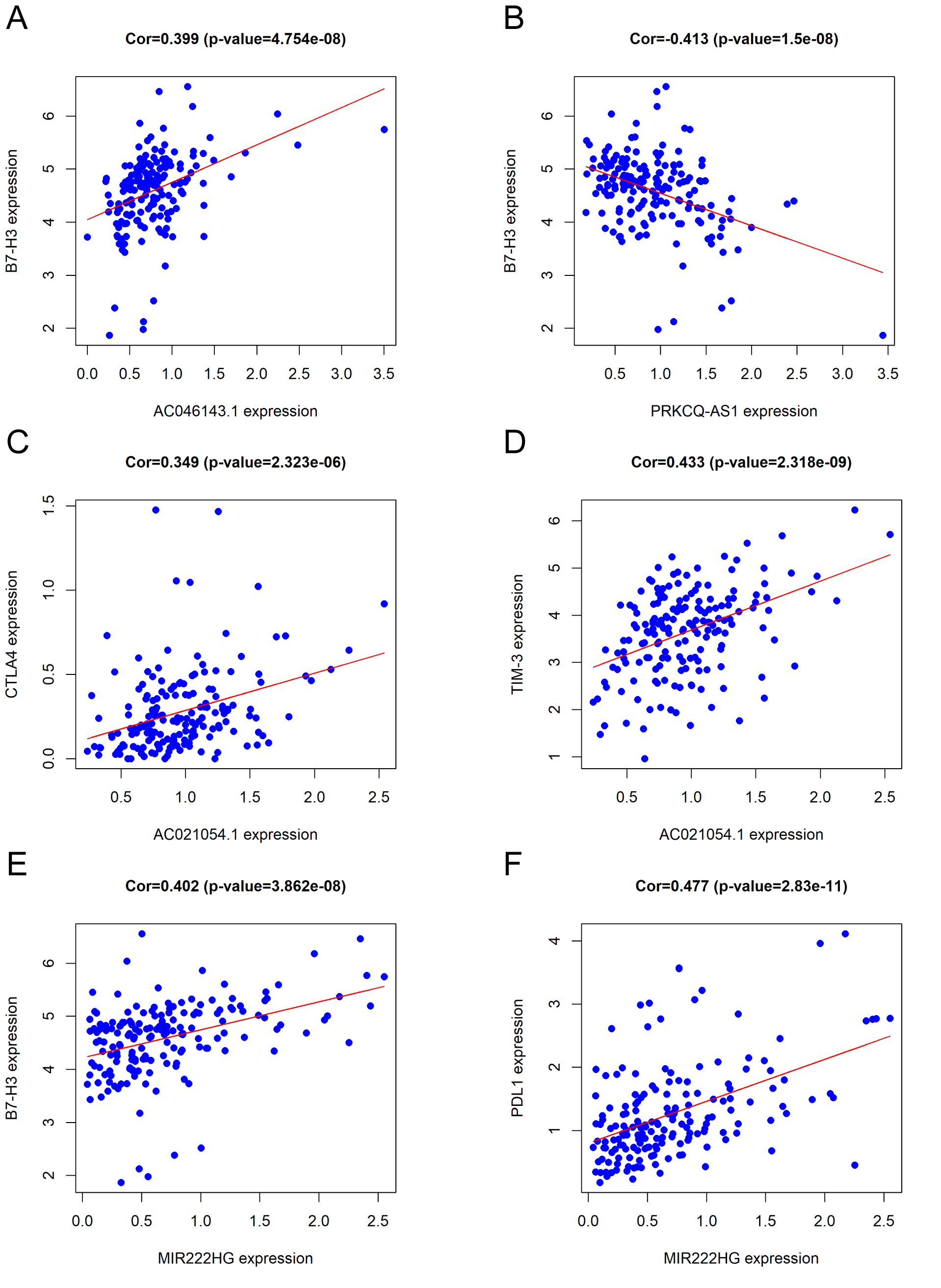

Supplement: Supplementary file 4 [file image4.tif]
