# Supplementary material for: A Five Immune-Related lncRNA Signature as a Prognostic Target for Glioblastoma
Source: Front Mol Biosci. 2021 Feb 16;8:632837. doi: 10.3389/fmolb.2021.632837 (PMC7921698; doi:10.3389/fmolb.2021.632837)
Supplement: Supplementary file 5 [file table1.docx]

| Characteristic | Training set (n=76) | Validation set (n=75) | Entire TCGA set (n=151) |
| --- | --- | --- | --- |
| Age |  |  |  |
| ≤50 | 16 | 20 | 36 |
| >50 | 60 | 55 | 115 |
| Sex |  |  |  |
| Female | 29 | 25 | 54 |
| Male | 47 | 50 | 97 |
| KPS |  |  |  |
| ≤70 | 14 | 18 | 32 |
| >70 | 44 | 39 | 83 |
| Unknown | 18 | 18 | 36 |
| Radiotherapy |  |  |  |
| No | 9 | 9 | 18 |
| Yes | 64 | 62 | 126 |
| Unknown | 3 | 4 | 7 |
| Chemotherapy |  |  |  |
| No | 11 | 13 | 24 |
| Yes | 61 | 51 | 112 |
| Unknown | 4 | 11 | 15 |

**Table S1** **Summary of patient demographics and clinical characteristic**
