# Supplementary material for: A Five Immune-Related lncRNA Signature as a Prognostic Target for Glioblastoma
Source: Front Mol Biosci. 2021 Feb 16;8:632837. doi: 10.3389/fmolb.2021.632837 (PMC7921698; doi:10.3389/fmolb.2021.632837)
Supplement: Supplementary file 6 [file table2.docx]

**Table S2 Univariate and multivariate Cox regression analysis of the five-lncRNA signature and molecular features**

| Variables | Univariate analysis | |  |  | Multivariate analysis | |  |
| --- | --- | --- | --- | --- | --- | --- | --- |
|  | HR | 95% CI of HR | p value |  | HR | 95% CI of HR | p value |
| Five-lncRNA risk model (high/low) | 1.424 | 1.267-1.599 | 2.73e-09 |  | 1.386 | 1.208-1.590 | 3.14e-06 |
| IDH1 (wild type/mutation) | 0.297 | 0.108-0.816 | 0.019 |  | 0.336 | 0.119-0.945 | 0.039 |
| MGMT (unmethylated/methylated) | 1.681 | 1.052-2.687 | 0.030 |  | 1.314 | 0.812-2.127 | 0.265 |
