# Supplementary material for: A Five Immune-Related lncRNA Signature as a Prognostic Target for Glioblastoma
Source: Front Mol Biosci. 2021 Feb 16;8:632837. doi: 10.3389/fmolb.2021.632837 (PMC7921698; doi:10.3389/fmolb.2021.632837)
Supplement: Supplementary file 7 [file table3.docx]

**Supplementary Figure 1.** The whole immune-lncRNAs co-expression networks **(A)**. Immune-lncRNAs co-expression networks regarding to the five identified immune-related lncRNAs **(B)**.

**Supplementary Figure 2.** Expression of five immune-related lncRNAs in the training set **(A)**, the validation set **(B)** and the entire TCGA set **(C)**. *P<0.05, **P<0.01, ***P<0.001

**Supplementary Figure 3.** The predictive performance comparison of the lncRNA signature (LncSig) with other lncRNA signature (ZhangSig, LiSig and PanSig) of 1-year OS (**A**) and 2-year OS (**B**). ROC, receiver operating characteristic; OS, overall survival.

**Supplementary Figure 4.** Correlation analysis of five lncRNAs with immune checkpoints, including AC046143.1 with B7-H3 **(A)**, PRKCQ-AS1 with B7-H3 **(B)**, AC021054.1 with CTLA4 **(C)**, AC021054.1 with TIM-3 **(D)**, MIR222HG with B7-H3 **(E)** and MIR222HG with PDL1 **(F)**.
